# Operative stabilisation versus non-operative management of mid-shaft clavicle fractures

**DOI:** 10.1051/sicotj/2022046

**Published:** 2022-11-25

**Authors:** Alla Vasanth Kumar, K. Ramachandra Kamath, Preetham Raj V. Salian, Sunil Lakshmipura Krishnamurthy, Rajendra Annappa, Ishwara Keerthi

**Affiliations:** 1 Department of Orthopaedics, Asram Medical College Eluru Andhra Pradesh India; 2 Department of Orthopaedics, Kasturba Medical College, Mangalore, Manipal Academy of Higher Education Manipal Karnataka 576104 India; 3 Clinical Fellow in Sports Injury and Arthroscopy, Department of Orthopaedics, Division of Sports Injury and Arthroscopy, St John’s Medical College Bangalore Karnataka 560034 India

**Keywords:** Midshaft clavicle fracture, Clavicle plate fixation, Conservative management

## Abstract

*Introduction*: Fractures of the mid-shaft clavicle are commonly encountered in clinical practice. These can be managed either by conservative method or operative by internal fixation. This study aims to compare the outcomes of conservative and operative management. *Methods*: Forty patients with displaced and comminuted mid-shaft clavicle fractures were included in the study, among which twenty-five patients were treated conservatively and 15 patients underwent surgery and were followed up for a period of 1 year. Time taken for union, functional outcome, complications, and patient satisfaction were compared. *Results*: In the non-operative group, 28% of the fractures took less than twelve weeks to unite, whereas in the operative group 60% of them took less than 12 weeks to unite. At the end of 1 year, there was no statistical difference in mean UCLA (University of California and Los Angeles) score and the mean DASH score of the non-operative group and operative group. There were more complications in the operative group. Re-operative rate in the operative group was 40%. Patient satisfaction was 80% in the non-operative group, whereas 48% of patients were satisfied in the operative group. *Conclusion*: Displaced and comminuted mid-shaft clavicle fractures treated conservatively have more advantages when compared to surgically treated fractures.

## Introduction

Fractures of the clavicle have an occurrence of about 2.6–4% of all fractures. More than 80% of the clavicle fracture occur in mid- shaft because of a narrow cross-section of bone and excess compressive forces of the shoulder [1, 2]. Various methods for managing these fractures are conservative, by immobilizing in a clavicular brace and arm sling and operative, by fixation with intramedullary nails or by anatomical locking plates. Conventionally, all mid-shaft clavicle fractures have been treated conservatively for ages, but now there is a change in trend from the conservative methods to a more surgical approach. Some studies favour surgical intervention while some are in favour of conservative management. There is no clear consensus on which mode of treatment is better, as both conservative and operative management have their own complications and advantages [3, 4]. There are few accepted indications for operative intervention in midshaft clavicle fractures as described in the literature which includes open fractures, polytrauma patients and fractures with neurovascular deficits which are excluded in most studies. In mid-shaft clavicle fractures, operative intervention is done to lessen the chances of nonunion, to gain early movements in active individuals and better early functional results [5]. The complications of non-operative treatment, which include non-union, malunion, altered shoulder mechanics, slow rate of healing, pressure effects of bump on brachial plexus, have encouraged the shift towards operative management. The advantages of operative management are reduced chances of nonunion and improved early functional results Though the operative treatment appears to be a good option, even this mode of treatment has its own complications, the common being the reoperation for implant removal [3–7]. Hence it is still under debate to advise surgery for all patients. The purpose of this study was to analyze the outcome in a series of patients with displaced and comminuted (type 2b Robinson) mid-shaft clavicle [8] fractures when treated conservatively and surgically, to compare which mode of treatment, either conservative or operative is more advantageous to the patient, based on the evaluation of clinical results in terms of time taken for union, functional outcome of the shoulder, complication rate and patient satisfaction. This study, which was conducted with direct patient contact, might help in giving some more evidence related to the management of mid-shaft clavicle fractures, particularly in the Indian set of population.

## Materials and methods

We conducted a prospective study on patients presenting to the Department of Orthopaedics in a tertiary care hospital. This study was an observational study. Informed consent was taken from all participants who were included in the study. The sampling method was convenience sampling.

Patients of age 18–60 years, with displaced (>1.5 cm) closed mid-shaft clavicle fracture (type 2b Robinson) were included in the study. Patients with an un-displaced clavicle fracture, fractures at the lateral end and medial end of the clavicle, open fractures, pathological fractures, and clavicle fractures associated with the humerus, scapula fractures, and neurological compromise were excluded from the study. Institutional Ethics committee approval was taken before starting the study. Inclusion and exclusion criteria for both operative and non-operative treatment were the same, as only displaced (>1.5 cm) and comminuted clavicle fractures (Robinson type 2b) [8] were included and treated as per patients’ informed decision, as no conclusion regarding which mode of treatment is advantageous to the patient was present at the start of the study. Patients were explained by the treating surgeon regarding the treatment options available, and the advantages and disadvantages of both conservative and surgical management, based on conclusions drawn in previous literature. Patients who were willing to conservative management were included in the non-operative group and patients who were willing for surgery were included in the operative group. No randomization was done. The study was an observational and prospective study.

Patients who opted for conservative management were treated with a figure of 8 clavicular braces which were applied and tightened to maintain clavicle reduction. The patient’s limb was immobilized in a sling and was advised to not lift weights and to continue to brace for 4 weeks. Those who consented to undergo surgery, after being explained about all modalities of management, underwent routine pre-operative investigations and were operated on with a pre-contoured locking compression plate.

*Post-op protocol*: Postoperative x-rays were taken, to study the alignment of fracture fragments. Gentle shoulder pendulum exercises, elbow and wrist mobilization were started at the end of 1 week. After two to three weeks, a gentle actively assisted range of motion of the shoulder was allowed with abduction limited to 80°. Active range of motion in all planes was started after 6 weeks, depending on the degree of the union at the fracture site.

**Figure 1:**
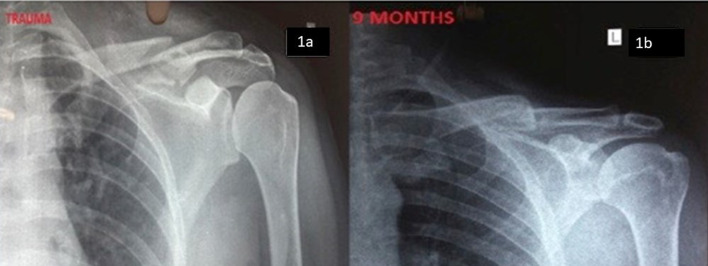
(a) Clavicle fracture, managed with brace (1b) Union with a good result after 9 months.

Follow-up was done at 6 weeks, 3 months, 6 months, 9 months, and 1 year. At every follow-up, the functional assessment was done based on scores calculated with the help of a Quick DASH questionnaire and UCLA to assess the function of the shoulder and x-rays were taken to check for implant position, malunion, radiological union while assessing non-union (those not united even at 9 months) and the time taken for the union was noted. The fracture was considered to be united on the basis of clinical findings (absence of fracture site tenderness, good functional range of motion) and radiological finding of a bridging callus, seen at the fracture site. Complications after conservative management were non-union, malunion (taking only symptomatic malunion, causing functional disability into consideration), and any neurological complications due to pressure symptoms of the bump. The complications in the operative group were hardware prominence, plate breakage, infection, decreased sensation at the infra clavicle region, non-union and malunion. Patient satisfaction was also assessed in terms of functional outcome, complications, cosmetics of scar and bump, and re-operative rate (Fig. 1).

**Figure 2:**
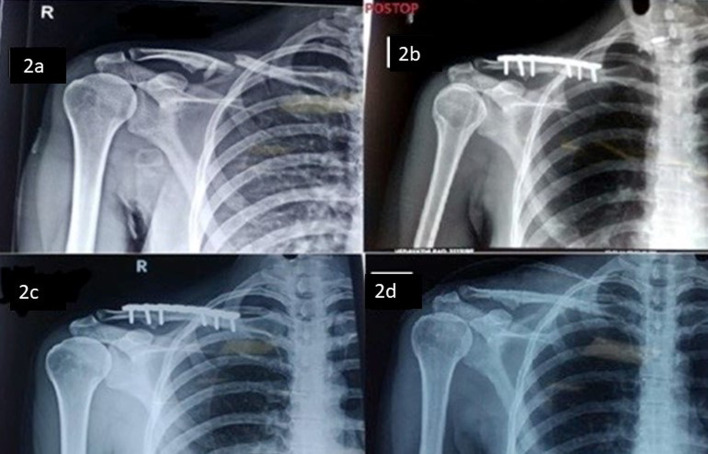
(a) Clavicle fracture, (b) postoperative x-ray after 3 months, (c) fracture union with good results and (d) after implant removal at 1 year.

Statistical data analysis was done by student unpaired *t*-test and Chi-square test and Fisher exact test. SPSS (Statistical Package for Social Science) version 17 was used, and *p* < 0.05 was considered significant (Fig. 2).

In this study, 40 patients with mid-shaft displaced clavicle fractures were studied, among which, 15 patients underwent surgery and 25 were managed conservatively. The mean age was 38 years. Among the 40 patients, 9 (22.5%) were females and 31 (77.5%) were males. Among the 40 patients, the mechanism of injury was due to road traffic accidents in 24 (60%), due to fall on an outstretched hand in 12 (30%), and due to sports injury in 4 (10%). According to Robinson’s classification, 12 fractures (30%) belong to 2B2 and 28 fractures (70%) belong to 2B1. Out of 40 patients, 29 (73%) patients had left-sided clavicle fractures and 11 (27%) patients had right-sided clavicle fractures (Table 1).

**Table 1:** Age distribution.

Age (years)	Non-operative		Operative		Total	
	Number (*N*)	%	*N*	%	*N*	%
30 and below	7	28	3	20	10	25
31–40	8	32	6	40	14	35
41–50	9	36	1	6.7	10	25
Above 50	1	4	5	33.3	6	15
Total	25	100	15	100	40	100

## Results

We studied the time for fracture union in both groups. Out of the 25 patients treated non-operatively, 7 (28%) patients’ fractures took less than 12 weeks to unite, whereas out of the 15 patients treated by operative means, 9 (60%) took less than 12 weeks to unite (Table 2).

**Table 2:** Fracture union time and nonunion.

Time for union	Non-operative (%)	Operative
8–12 weeks	7 (28%)	9 (60%)
>12 weeks	16 (64%)	5 (33.33%)
Nonunion	2 (8%)	1 (6.7%)
Total	25	15

### Functional outcomes

Functional assessment of individual groups was done based on UCLA and DASH scores. The mean UCLA score of both groups shows an increase in scores with every follow-up as illustrated (Table 3).

**Table 3:** Average UCLA scores at each follow-up.

UCLA scores	Group	*N*	Mean	Std deviation	Median (IQR)	Mann-Whitney test *Z*-value	*P*
3 months	Non-operative	25	21.76	6.57	20 (19–27)	0.35	0.726
	Operative	15	19.87	8.47	20 (18–27)		NS
6 months	Non-operative	25	26.76	6.67	26 (22.5–33)	0.01	0.989
	Operative	15	26	8.19	27 (16–33)		NS
9 months	Non-operative	25	31.12	4.67	33 (30–35)	0.37	0.709
	Operative	15	29.8	6.19	32 (25–35)		NS
1 year	Non-operative	25	34.16	2.32	35 (35–35)	1.23	0.219
	Operative	15	32.8	3.95	35 (28–35)		NS

The mean DASH scores in both groups show a decrease in disability with every follow-up as illustrated. There is no significant functional difference between the two groups at the end of 1 year (Table 4).

**Table 4:** Average DASH scores at each follow-up.

DASH scores	Group	*N*	Mean	Std deviation	Median (IQR)	Mann-Whitney test *Z*-value	*P*
3 months	Non-operative	25	35.18	14.23	42.2 (20.5–43.7)	0.35	0.726
	Operative	15	38.94	20.42	42.2 (20.5–60.4)		NS
6 months	Non-operative	25	21.16	12.37	25.5 (9.2–29.5)	0.01	0.989
	Operative	15	25.11	17.41	29.59 (9.2–29.5)		NS
9 months	Non-operative	25	4.63	6.07	2.5 (2.3–2.65)	0.37	0.709
	Operative	15	9.18	12.19	2.5 (2.3–22.7)		NS
1 year	Non-operative	25	0.28	0.76	0 (0–0)	1.23	0.219
	Operative	15	1.37	3.51	0 (0–2.3)		NS

### Complications

**Figure 3:**
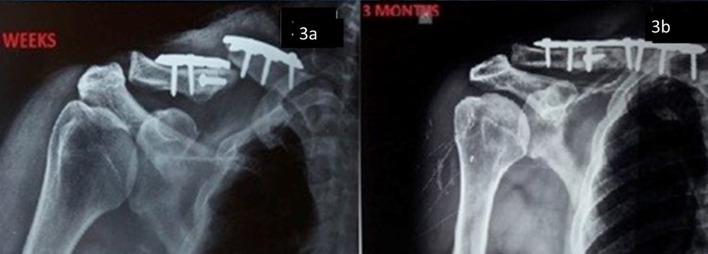
Complications after plating (a) Broken plate at 6 weeks, (b) postoperative x-ray 3 months after revision surgery.

**Figure 4:**
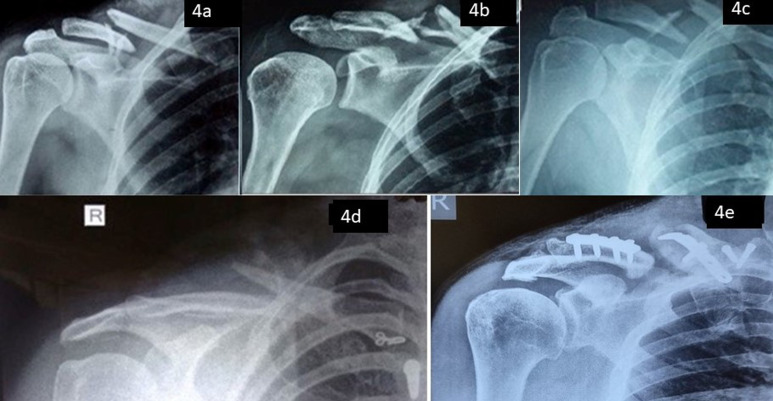
(a)–(c) Preoperative radiographs of fractures with non-union (4d) Nonunion after non-operative treatment (b) Nonunion after operative management.

In the non-operative group, 2 patients (8%) had non-union and almost all patients had malunion of varying degrees, but considering only fractures with significant malunion causing functional variance and non-union, only 5 patients (20%) had complications compared to the operative group, where 9 patients (60%) had complications, among which 2 (8%) had non-union and 2 (8%) had malunion. The re-operative rate was 40% in the operative group (Figs. 3 and 4).

**Figure 5:**
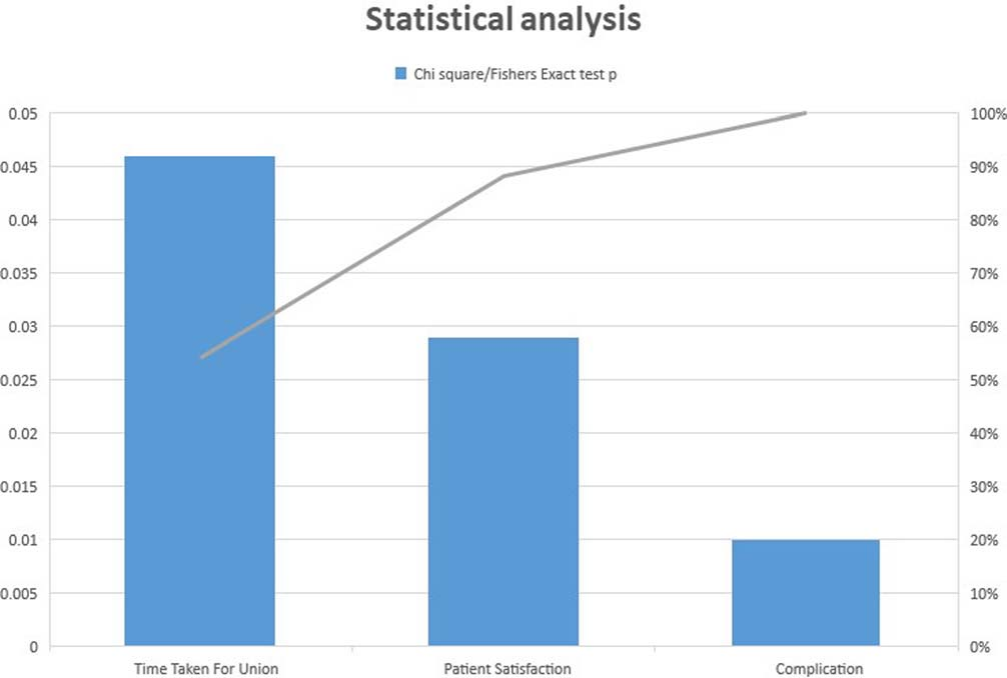
Patient satisfaction and statistics.

In the operative group, some patients experienced more than one complication. Taking into consideration the number of patients, who suffered complications in both operative (9) and non-operative (5), a significant difference was obtained (0.010). There were 11 patients with a fracture displacement of greater than 2 cm in the study, who were treated conservatively among which, 3 patients had complications (1 non-union, two with functionally significant malunion). With respect to patient satisfaction, in the non-operative group, 20 (80%) patients were satisfied compared to 8 (53.33%) in the operative group (Fig. 5).

## Discussion

Management of displaced mid-shaft clavicle fracture is still controversial. The main aim of the surgeon is to get good reduction with minimal complications, early union and good functional outcome, and better patient satisfaction. Both conservative and operative management has been recommended when literature is reviewed. From this study on mid-shaft clavicle fractures, we have found that the average time taken for fracture union in patients who underwent surgery is significantly lower than in patients treated conservatively, with complications being significantly higher in the operative group. The functional outcome shows no significant difference in the patients treated by operative and non-operative methods. We found that the patient satisfaction rate is higher in the non-operative group than in the operative group.

The limitations of our study were the small sample size and lesser follow-up time.

Traditionally, a treatment plan for all midshaft clavicle fractures is the non-operative approach. Clavicle fractures with a displacement greater than 2 cm had higher rates of non-union and malunion, particularly fractures with comminution. They also had inferior functional scores, due to patient dissatisfaction and decreased abduction strength [9].

According to recent literature, mid-shaft clavicle fractures treated surgically healed at a faster rate than those treated conservatively [10]. Though surgical treatment of clavicle fractures is gaining popularity, it has also been found that more complications are being identified for surgical treatment).The common complications are numbness at the incision site due to supraclavicular nerve injury in plating and pain at the surgical site of the scar, cosmetic unhappiness, and implant irritation [11, 12]. To achieve the best results, the surgeon should select a suitable patient for surgery in order to avoid unnecessary surgical intervention and hence avoid complications and reduce the burden on the patient. In an active individual surgical management is advised for early return to activities and better short-term functional outcomes [5].

Plate fixation for displaced midshaft clavicle fractures resulted in a lower rate of malunion and nonunion compared to sling immobilization. Though surgical procedures have low rates of non-union and malunion, implant-related complications are very high (9–64%). Implant removal was the most common second surgery as described in these studies [13–15].

In a systematic review of clavicle fractures by Ban et al. [14] it was found that functional outcome was better in surgically treated fractures, with the complication rate higher in the conservative group (47%) compared to the surgically treated group (30%). Whereas in this study only 20% of the patients treated conservatively had a significant complication, considering only functionally significant malunion and non-union, while 60% of patients who underwent surgery had complications (*p*-value = 0.010). Faldini et al. [15], in their study on conservative treatment of clavicle fractures, reported that non-operative treatment achieves good results, similar to operative treatment, in terms of time taken for union, patient satisfaction and functional outcome without any surgical complications. Similarly, in this study, there is no statistically significant functional difference between both the groups with patient satisfaction significantly higher in the non-operative group (*p*-value = 0.029) and a higher chance of complications in the operative group (*p*-value 0.010).

In 2013, the Cochrane Collaboration [16] suggested treatment options for mid-shaft clavicle fractures which were decided on an individual patient basis, after taking careful consideration of the relative benefits and harms of each intervention and the preference of the patient. In our study, 80% of the conservatively treated patients were satisfied, whereas 46% of the operated patients were satisfied, which is similar to the study by Digeorgi et al. [17], who found 71.8% of the subjects to be satisfied with conservative treatment. Conservative management is associated with the absence of unnecessary soft tissue and periosteal stripping, and more time is taken to unite radiologically but the return to clinical functionality was similar to that of operated cases. Ninety eight percent of those managed conservatively had malunion as a complication, but it had no functional significance, neurological or pressure symptoms and the initially occurring large bump decreases in size with time due to remodelling and hence has no cosmetic significance. Whenever surgical modality is planned for management patients should be counselled for expected results and the need for revision surgery especially for implant removal. In conservative management, patients should be made aware of rates of non-union and difficulty in its management [18].

These results illustrate that even though the time taken for union is less in the operative group, there is no significant functional difference between the two groups, with the presence of a higher complication rate in the operative group, taking into consideration the presence of non-union and malunion even after treatment by means of surgery. Conservative treatment may be preferred over operative methods in view of decreased significant complications, similar functional outcomes compared to the operative group, better patient satisfaction, better outcome, even in fractures displaced greater than 2 cm, and prevention of unnecessary intervention, particularly in developing countries where affordability is an issue. Even fractures displaced greater than 2 cm unite properly if good bracing and strict immobilization are maintained.

## Conclusion

Displaced and comminuted mid-shaft clavicle fractures treated by surgery have early union and a higher complication rate. Conservatively managed fractures have similar functional outcomes and better patient satisfaction.
